# A zebrafish drug screening platform boosts the discovery of novel therapeutics for spinal cord injury in mammals

**DOI:** 10.1038/s41598-019-47006-w

**Published:** 2019-07-19

**Authors:** Diana Chapela, Sara Sousa, Isaura Martins, Ana Margarida Cristóvão, Patrícia Pinto, Sofia Corte-Real, Leonor Saúde

**Affiliations:** 1 TechnoPhage, SA, Av. Prof. Egas Moniz, 1649-028 Lisboa, Portugal; 2 Instituto de Medicina Molecular e Instituto de Histologia e Biologia do Desenvolvimento, Faculdade de Medicina da Universidade de Lisboa, 1649-028 Lisboa, Portugal; 3 Instituto de Medicina Molecular, Faculdade de Medicina da Universidade de Lisboa, 1649-028 Lisboa, Portugal

**Keywords:** Spinal cord diseases, Phenotypic screening, Neurodegenerative diseases

## Abstract

Spinal cord injury (SCI) is a complex condition, with limited therapeutic options, that results in sensory and motor disabilities. To boost discovery of novel therapeutics, we designed a simple and efficient drug screening platform. This innovative approach allows to determine locomotor rescue properties of small molecules in a zebrafish (*Danio rerio*) larval spinal cord transection model. We validated our screening platform by showing that Riluzole and Minocycline, two molecules that are in clinical trials for SCI, promote rescue of the locomotor function of the transected larvae. Further validation of the platform was obtained through the blind identification of D-Cycloserine, a molecule scheduled to enter phase IV clinical trials for SCI. Importantly, we identified Tranexamic acid and further showed that this molecule maintains its locomotor recovery properties in a rodent female contusion model. Our screening platform, combined with drug repurposing, promises to propel the rapid translation of novel therapeutics to improve SCI recovery in humans.

## Introduction

Spinal cord injury (SCI) is a permanent and chronic condition in humans that leads to motor, sensory and autonomic dysfunction. Treatment options, either standard of care or experimental, have met limited success in providing severely afflicted patients with good neurological and functional recovery^1^.

SCI triggers apoptosis of neurons and oligodendrocytes, leading to demyelination^2^. In addition, this primary damage severs axonal tracts and blood vessels. The disruption of the vasculature leads to parenchymal haemorrhage that cause further cell death and allows the extravasation of immune cells^3,4^. The inflammatory reaction has a critical role in the secondary damage by inducing a second wave of apoptosis^5^. Additionally, persistent inflammation was associated with glial scarring formed by reactive astrocytes and pericytes that proliferate and migrate towards the inflamed lesion site^6–8^. Although the glial scar provides structural support, it also creates an inhibitory environment for regenerating axons, thus preventing the re-enervation of the original targets^9–11^.

The majority of molecular therapies for SCI aims to protect neurons from secondary cell death, to promote axonal regrowth and to enhance nerve conduction. One of such promising therapies is the use of Riluzole, a benzothiazole, shown to have neuroprotective effects that seem to diminish neurological tissue destruction and to ameliorate functional recovery in mammalian SCI models. This molecule is now in a phase II/III multi-centre clinical trial^10,12^. Another promising molecule is Minocycline, a semi-synthetic tetracycline derivative that has been clinically available as an antibiotic and anti-inflammatory drug. Recently, the intravenous administration of Minocycline showed to reduce cell death and improve hindlimb function in mammal SCI models^13,14^.

Given the complex nature of SCI, it is likely that only multiple-target therapies will achieve significant functional recovery^1,15^. The possibility of exploring a considerably large number of new compounds for SCI, in an unbiased manner, would be highly valuable. Zebrafish larvae are an established model for medium-high throughput drug screens^16–18^. Recent studies have begun to elucidate the degree of physiologic conservation between zebrafish and humans, which share 82% of disease associated targets and a large number of drug metabolic pathways^19^. Importantly, several compounds discovered in zebrafish screens have been shown to exhibit similar effects in rodent models and humans^17^.

In what concerns SCI, it has been shown that the larva and adult zebrafish spinal cords also undergo haemorrhage, inflammation and apoptosis leading to cell loss and demyelination. Importantly, and in clear contrast with mammals, these cellular processes are rapidly resolved in zebrafish^20–22^. The regenerative capacity of the zebrafish spinal cord also relies on the ability of producing new neurons from neural stem cells that reside in the ependymal region to replace the lost ones and also to the formation of a glial bridge that helps the regrowth of severed axons. The outcome is a clear and rapid recovery of the motor function after injury^23–27^. This contrasts with the situation in mammals where the formation of a fibrotic/glial scar inhibits the regrowth of axons from neurons that have eventually survived the lesion. Clearly mammals and zebrafish display different outcomes upon SCI despite sharing a considerable number of molecular and cellular pathways^28,29^.

It is therefore realistic to expect that compounds identified through zebrafish screens will be useful to reactivate and modulate similar pathways in mammals. In this study, we describe a simple, fast and automated phenotypic FDA-approved drugs screen in a whole-animal *in vivo* context, using a transected spinal cord larval model of SCI. Importantly, we were able to validate our zebrafish larval screening with Dopamine, a molecule that promotes motor neuron regeneration in SCI animal models^30,31^ and also Riluzole and Minocycline, two promising molecules that entered clinical trials for SCI indication^1,12–14^. Additionally, we blindly identified D-Cycloserine, a molecule scheduled to enter phase IV clinical trials for SCI indication, further validating our screening platform.

We identified, for the first time to our knowledge, Tranexamic acid, a synthetic analogue of lysine known to inhibit fibrinolysis^4,32^ as a molecule with motor recovery properties in zebrafish larvae. We went on to test this molecule in a rodent T9 contusion model of SCI and showed its cellular and locomotor efficacy.

Here, we present a full proof-of-concept of a transected spinal cord zebrafish larval model for the search of new therapeutics for SCI. This new approach promises to accelerate the period from drug discovery to clinical use.

## Results

### Zebrafish larvae with transected spinal cords fully recover their locomotor function after 6 days

As zebrafish larvae can regenerate the spinal cord after an injury, our screen was designed to identify small molecules that would accelerate the regenerative process. Therefore, it was crucial to define precisely the time-course of events after spinal cord injury (SCI) in our experimental setting.

**Figure 1:**
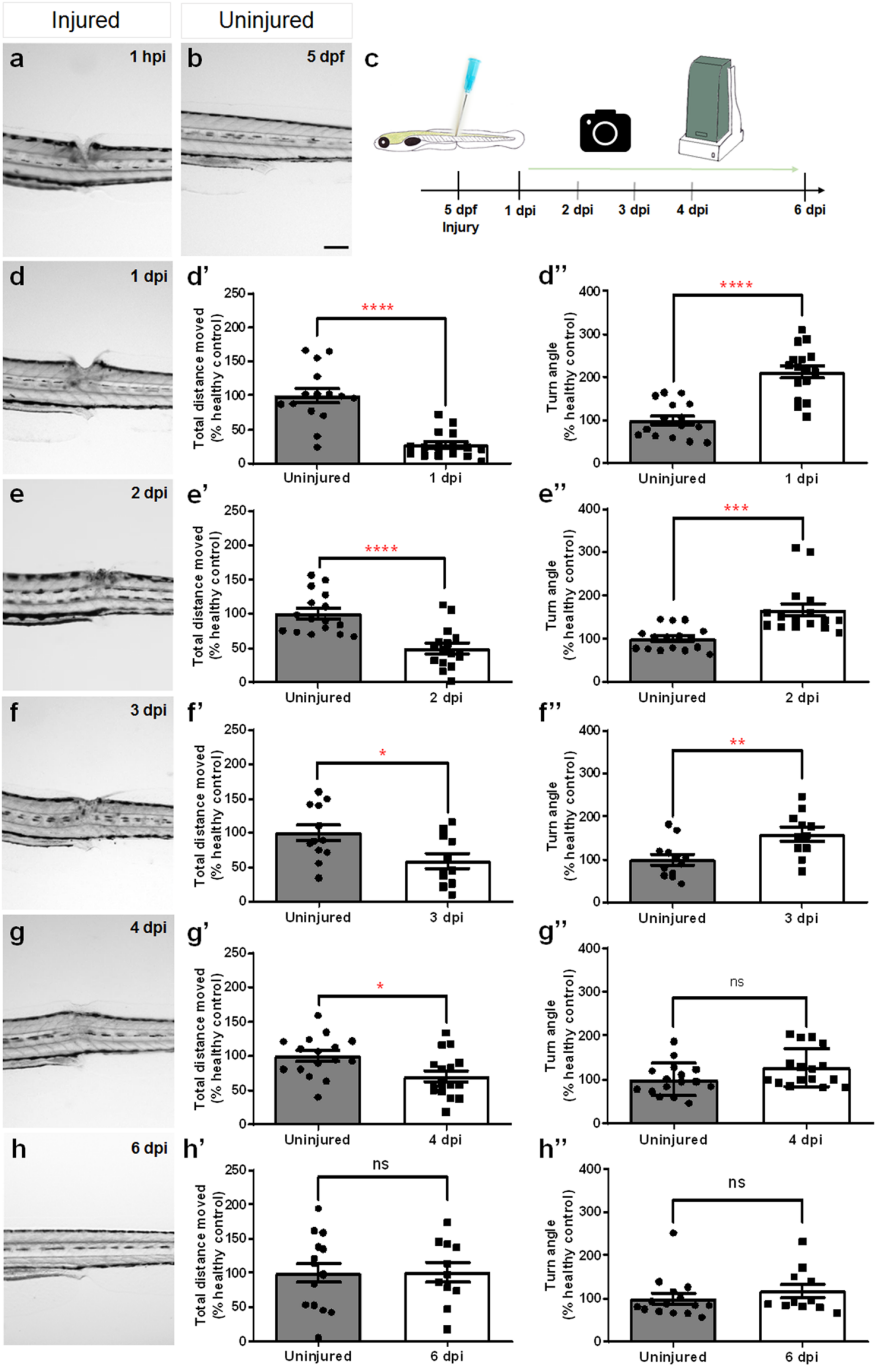
Temporal profile of tissue regeneration and locomotor recovery upon spinal cord transection in 5 dpf zebrafish larvae. (**b**) Brightfield image of the trunk/tail area of an injured 5 dpf larva. (**c**) Experimental design for the analysis of the temporal profile of tissue regeneration and locomotor recovery. (**a**,**d**–**h**) Brightfield images of the recovery of zebrafish larvae with a lesion in the dorsal trunk area from 1 hpi to 6 dpi. (**d’**–**h’**) Total distance moved and (**d”**–**h”**) turn angle parameters of injured larvae and age-matched uninjured controls at 1 dpi (n = 16 uninjured larvae, n = 16 injured larvae), 2 dpi (n = 16 uninjured larvae, n = 16 injured larvae), 3 dpi (n = 12 uninjured larvae, n = 11 injured larvae), 4 dpi (n = 16 uninjured larvae, n = 16 injured larvae) and 6 dpi (n = 15 uninjured larvae, n = 11 injured larvae). dpf_days-post-fertilization, hpi_hours-post-injury. Mean ± s.e.m. of one experiment is presented. ns - not significant, *p < 0.05, **p < 0.01, ***p < 0.001 and ****p < 0.0001, Student’s t-test with Welch’s correction. Scale bar: 200 µm.

We performed a complete spinal cord transection using 5 days-post-fertilization (dpf) *hb9*:GFP larvae at the level of the anal pore, leaving the notochord and major blood vessels intact. Complete transection was verified visually by confirming a complete gap between rostral and caudal spinal cord stumps (Fig. 1a) and physiologically by lack of touch response caudal to the injury site (data not shown).

Wound closure and recovery of the tissue, as well as locomotor function (total distance moved and turn angle) were assessed *in vivo* from 1 to 6 days-post-injury (dpi) using a stereoscope and an automated video tracking system, respectively. After an injury, the wound closed quickly (within 48 hours) (Fig. 1e) and the tissues seemed to be completely recovered within 6 days (Fig. 1h). At each post-injury time point from 1 to 4 dpi, injured larvae showed statistically significant locomotor impairments when compared with uninjured zebrafish larvae, as measured by total distance moved (1 dpi, *t* = 6.631, *p* < 0.0001; 2 dpi, *t* = 4.781, *p* < 0.0001; 3 dpi, *t* = 2.564 *p* = 0.0181 and 4 dpi, *t* = 2.678, *p* = 0.0119) and turn angle (1 dpi, *t* = 6.294, *p* < 0.0001; 2 dpi, *t* = 4.122, *p* = 0.0005; 3 dpi, *t* = 2.990 *p* = 0.0074 and 4 dpi, *t* = 1.947, *p* = 0.0612) for 90 minutes, under 10 minutes light-dark cycles (Fig. 1d’–g’,d”–g” and Supplementary Table S1). After 6 recovery days, injured larvae significantly regained their locomotor function to the same levels of the uninjured group, as revealed by total distance moved (*t* = 0.07084, *p* = 0.9441) and turn angle (*t* = 0,8260, *p* = 0.4181) indicators (Fig. 1h’,h” and Supplementary Table S1).

### Glial bridge formation and motor neuron local regeneration are extremely quick at the larval stage

To examine the cellular dynamics recovery, we focused our attention on two critical events, the formation of the glial bridge and the regeneration of motor neurons at the injury site. We were able to evaluate both in the same larvae using a GFAP antibody staining in *hb9*:GFP transgenics.

**Figure 2:**
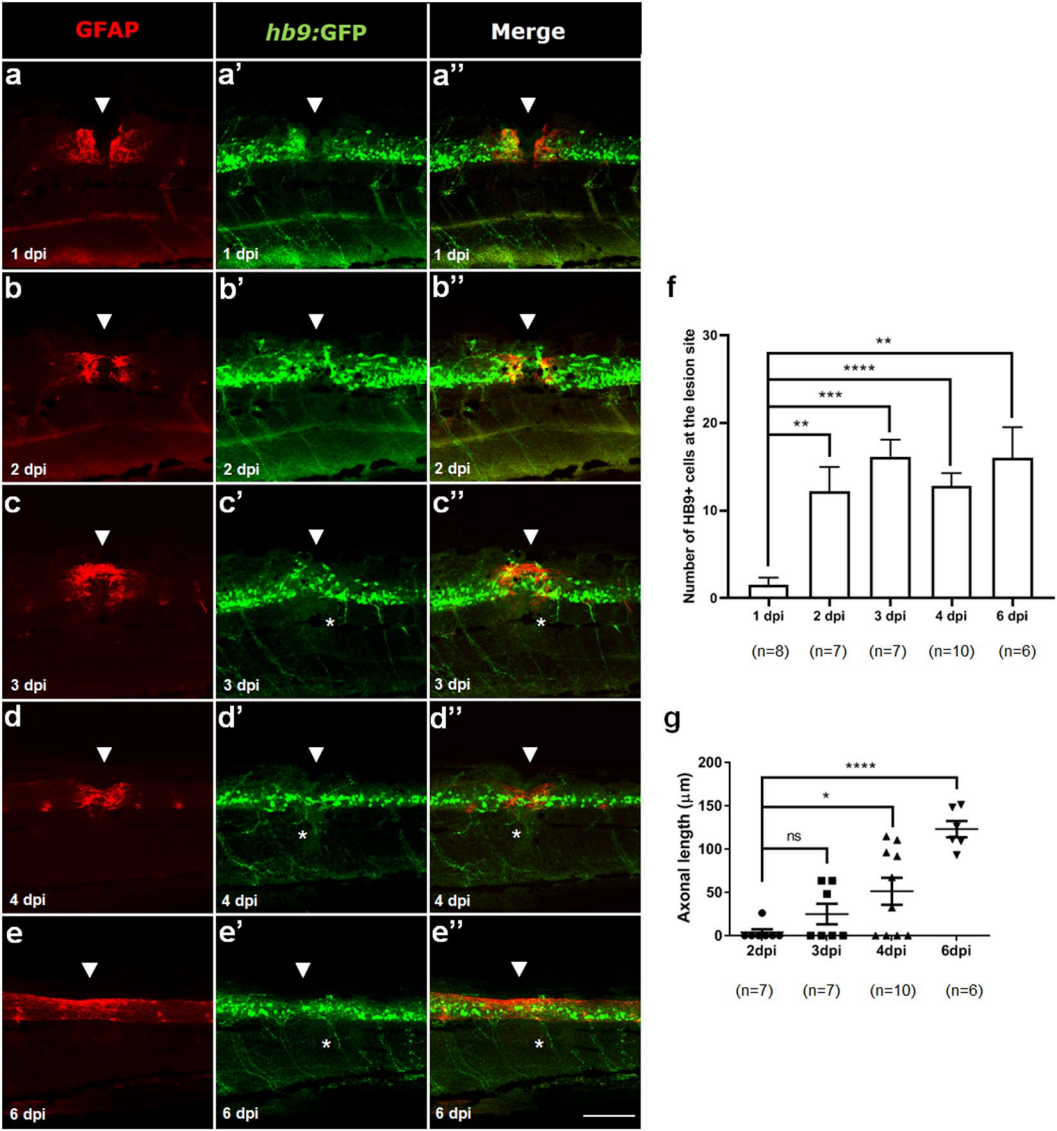
Glial bridge formation and motor neurons regeneration after SCI in 5 dpf larvae. Labeling pattern of *Hb9*:GFP transgenic larvae with double immunohistochemistry against GFAP (red) to reveal the glial bridge (**a**–**e**) and against GFP (green) to reveal HB9^+^ motor neurons (**a’**–**e’**) from 1 to 6 dpi. (**a”**–**e”**) Merged channels for each time point. White arrowheads show the lesion site and asterisks highlight the regenerating HB9^+^ peripheral axons. Rostral side is to the left and dorsal side is up. Number of HB9^+^ motor neurons (**f**) and length of HB9^+^ peripheral axons (**g**) at the lesion site from 1 to 6 dpi. SCI_spinal cord injury, dpf_days-post-fertilization, hpi_hours-post-injury Mean ± s.e.m. of one experiment is presented. ns - not significant, *p < 0.05, **p < 0.01, ***p < 0.001 and ****p < 0.0001, Student’s t-test with Welch’s correction. Scale bar: 100 µm.

At 1 day after complete transection, GFAP expression accumulated at spinal cord stumps without bridging and no motor neurons were visible at the lesion site (Fig. 2a–a”). Between 2–3 dpi, injured larvae started to form a glial bridge (Fig. 2b,c). At these time points, a significant increase in the number of HB9^+^ motor neurons was also visible at the lesion site (Fig. 2b–c”,f). In addition, we also found an evident regrowth of HB9^+^ peripheral axons at the lesion site between 3–4 dpi (Fig. 2c’,c”,d’,d”,f,g) that was fully re-established by 6 dpi (Fig. 2e’,e”).

### A phenotypic-based screen identifies compounds with locomotor rescue properties in a zebrafish larval transected spinal cord injury model

To select candidate compounds capable of rescuing motor impairments in zebrafish larvae with complete transected spinal cords, we developed a phenotypic assay that enabled the screening of a pool of small molecules from an FDA approved chemical compounds library.

We chose to perform the injury at the larval stage of 5 dpf, a time-point that coincides with a plateau of neurogenesis. This would then imply that beyond this time-point any neurogenic process would require reactivation of a regenerative program^33^. Moreover, a zebrafish larva at 5 dpf is anatomically mature making it a more suitable model of an adult paradigm.

**Figure 3:**
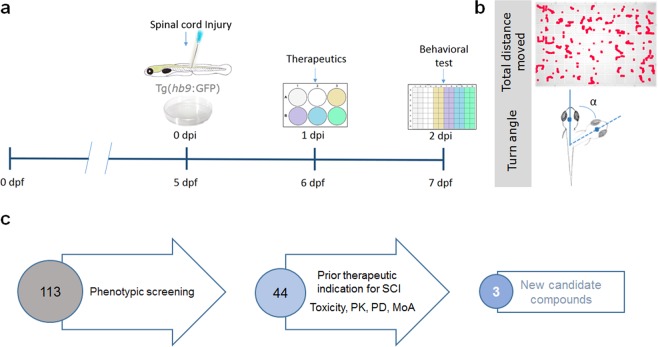
Schematic representation of the platform developed for the selection of novel compounds with SCI recovery properties. (**a**) The protocol of the phenotypic screening starts with the spinal cord transection of 5 days-post-fertilization (dpf) Hb9:GFP transgenic zebrafish larvae. The next day (1 dpi), the transected larvae are transferred into a 6-well plate. The therapeutics are added to the medium and zebrafish larvae are incubated for 24 hours. At 2 dpi, the behavioural analysis is conducted in a 96-well plate with a video tracking system. (**b**) Total distance moved and turn angle are used as indicators of locomotor function. (**c**) Schematic representation of the sequential criteria steps that led to the identification of the 3 potential new therapeutics for SCI.

In this phenotypic assay, we blindly administered the chemical compounds (25 µM) the day after injury (Fig. 3a). Behavioural assessment was carried out 24 hours later (i.e. at 2 dpi), a time-point where full cellular and motor recovery are not expected, as previously shown (Figs 1e–e” and 2b–b”). Total distance moved and turn angle were used as indicators of locomotor function (Fig. 3a,b).

In each experiment, 4 experimental conditions were generated: (1) larvae without transection + vehicle (uninjured control); (2) larvae with transection + vehicle (untreated control); (3) larvae with transection + positive control; (4) larvae with transection + small molecule from the library. The small molecules were first selected if there was a statistically significant improvement of total distance moved and/or turn angle parameters assessed in 3 independent experiments.

**Figure 4:**
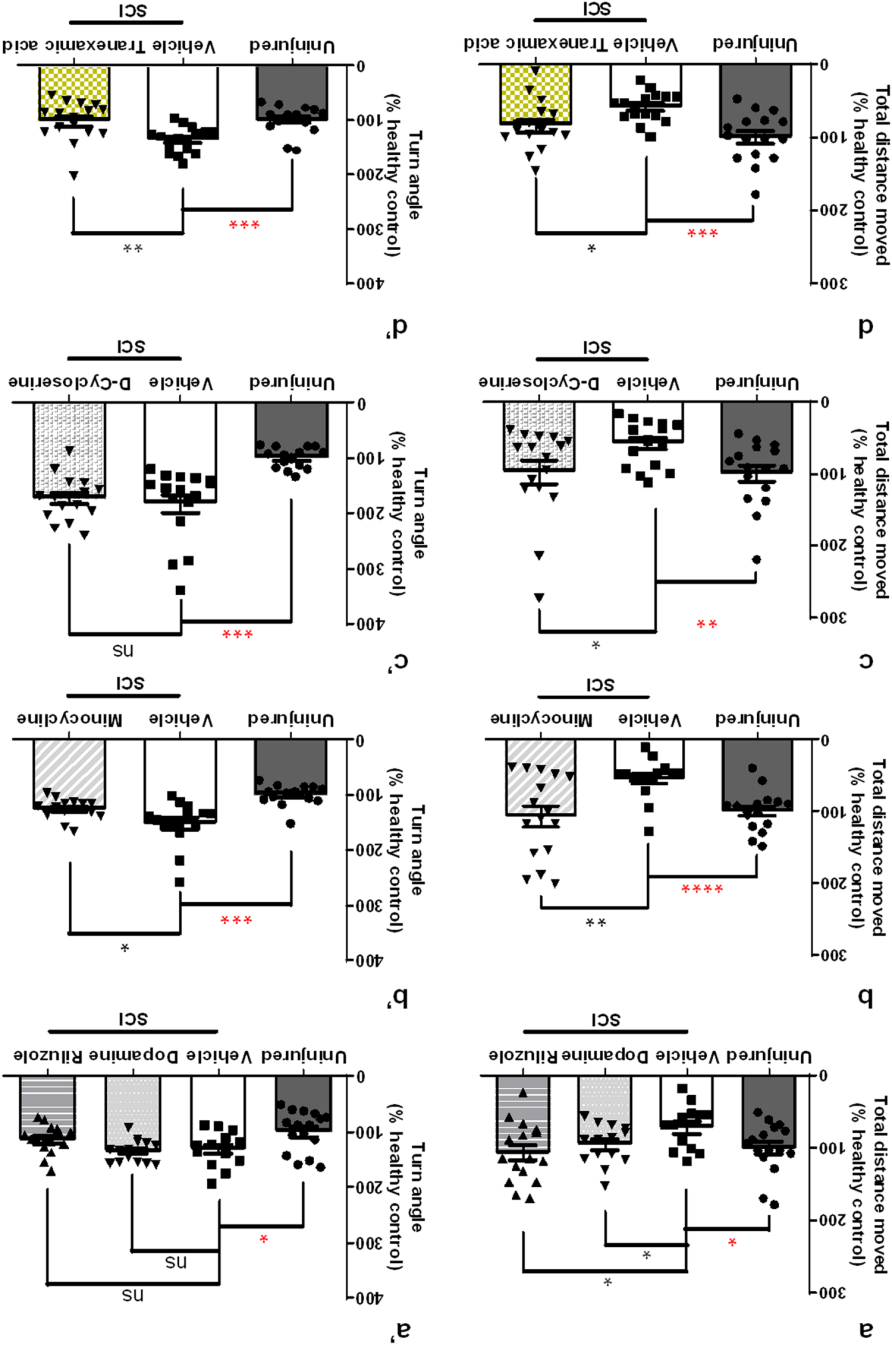
Rescue of motor impairments by different pharmaceutical classes of molecules in a transected zebrafish larval model of SCI. Locomotor performance depicted from total distance moved (**a**,**b**) and turn angle (**a’**,**b’**) in transected zebrafish larvae treated with 30 µM of Dopamine (n = 14 larvae), 0,5 µM of Riluzole (n = 16 larvae) and 25 µM of Minocycline (n = 16 larvae) as compared to vehicle treated injured larvae (n = 13–15 larvae). Locomotor performance depicted from total distance moved (**c**,**d**) and turn angle (**c’**,**d’**) in transected zebrafish larvae treated with D-Cycloserine (n = 16 larvae) and Tranexamic acid (n = 16 larvae), both picked from the library of FDA approved chemical compounds, as compared to vehicle treated injured larvae (n = 16 larvae). All percentages are relative to the mean of vehicle treated uninjured larvae (healthy control, n = 16 larvae). Mean ± s.e.m. of four independent experiments is presented. ns - not significant, *p < 0.05, **p < 0.01, ***p < 0.001 and ****p < 0.0001, Student’s t-test with Welch’s correction.

To validate the robustness of our screen, we tested the positive controls Minocycline (25 µM), Dopamine (30 µM) and Riluzole (0,5 µM) as they were previously known to rescue, to a certain extent, motor impairments in SCI animal models^1,12–14,30,31^. Indeed, we found that Dopamine, Riluzole and Minocycline administration resulted in the recovery of both total distance moved and/or turn angle parameters when compared to administration of vehicle (Fig. 4a–b’ and Supplementary Table S2). We decided to use Minocycline as the positive control, in all the independent experiments of the screen, as we demonstrated that this drug has a therapeutic effect when used with the same concentration as the one used in the screen.

From the initial pool of 113 molecules screened with our platform, we identified 44 small molecules that rescued motor impairments in injured zebrafish larvae. While during the screen, the molecules were blindly administered and selected, thereafter, we identified the chosen molecules to narrow down the selection based on defined exclusion criteria (Fig. 3c). 41 of the small molecules selected during the screen were excluded because one of the following criteria: patented or with reported therapeutic indication for SCI; do not cross the blood-brain-barrier; and with major reported toxicity (Fig. 3c). D-Cycloserine was one of such cases. It was blindly selected during the screen for rescuing motor impairments (total distance, *t* = 2.162, *p* = 0.0420) in larvae with transected spinal cords (Fig. 4c–c’ and Supplementary Table S2) and will enter in phase IV clinical trials for SCI indication. This result is particularly relevant as a proof-of-concept for the screen described here.

Our screening protocol allowed us to select 3 new candidate compounds with therapeutic properties for rescuing locomotor impairments upon SCI (Fig. 3c). One of the small molecules that showed to be particularly promising for further experiments was Tranexamic acid (total distance, *t* = 2.576, *p* = 0.0164; turn angle, *t* = 3.111, *p* = 0.0046; Fig. 4d–d’ and Supplementary Table S2), a synthetic analogue of lysine, which inhibits fibrinolysis and is used in the first hours after a traumatic haemorrhagic shock^4,32^. Moreover, we found a significant increase in the number of HB9^+^ motor neurons at the lesion site at 2 dpi in larvae treated with Tranexamic acid (t = 2.518, *p* = 0.0459, Supplementary Fig. S1a–c).

### Tranexamic acid improves behavioural scores following contusion spinal cord injury in a rodent model

**Figure 5:**
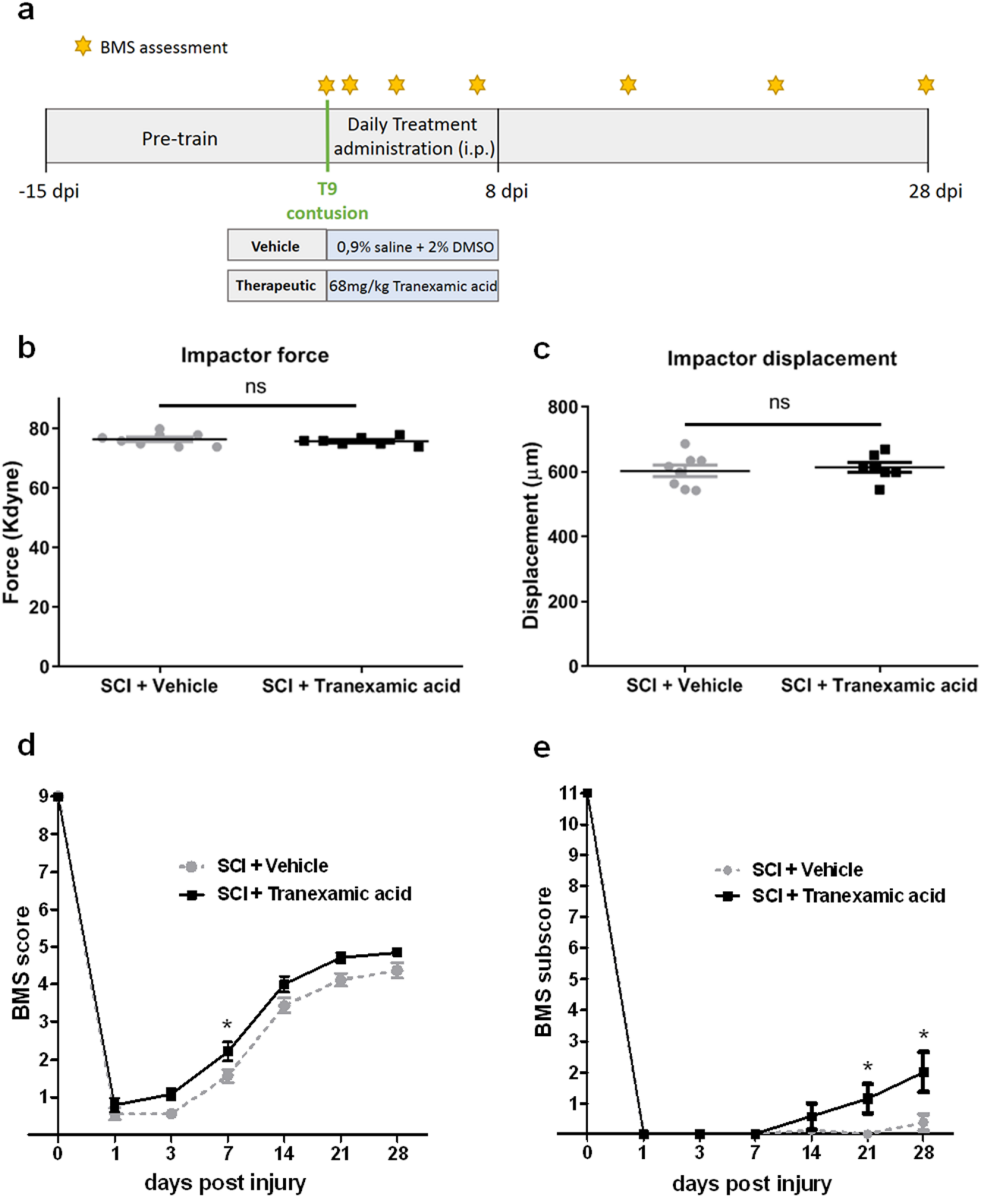
Locomotor recovery after T9 contusion in a rodent SCI model. (**a**) Experimental design of the Tranexamic acid efficacy test in a T9 contusion rodent model of SCI. (**b**) Impact force (kilodynes) imparted on the spinal cord during SCI (ns - not significant, t student test with Welch’s correction). (**c**) Displacement (μm) of the impactor tip upon contact with the spinal cord during T9 contusion (ns - not significant, t student test with Welch’s correction). (**d**) BMS scores of Tranexamic acid-treated mice compared with vehicle-treated mice from 1 to 28 dpi. (**e**) BMS subscores of Tranexamic acid-treated mice compared with the vehicle-treated mice from 1 to 28 dpi. Mean ± s.e.m. of one experiment is presented. *p < 0.05, Two-way repeated measures ANOVA followed by Bonferroni’s post-hoc correction. n = 8 for SCI+ Vehicle and n = 7 for SCI+ Tranexamic acid.

To validate the effect of Tranexamic acid as a therapeutic for SCI indication in mammals, we tested this molecule in a non-regenerative model. We used adult C57BL/6 female mice that were 12 weeks old to perform a T9 contusion using an Infinite Horizon (IH) Impactor. Mice were habituated to behavioural apparatus and were injured with a moderate-severe contusion injury (75 kdynes) (Fig. 5a).

Mice were randomly distributed to each experimental group (SCI+ vehicle and SCI+ Tranexamic acid). Both vehicle and Tranexamic acid were administered via intraperitoneal (i.p.) injection, beginning 1 hour after injury, and then daily until 8 days after injury (Fig. 5a). There were no differences in injury force or displacement applied by the IH Impactor between experimental groups (Fig. 5b,c). The BMS scores and BMS subscores were measured for 28 days (Fig. 5a,d,e). The BMS scores in the Tranexamic acid-treated mice and in the vehicle-treated mice increased after 1 dpi and reached a plateau at 28 dpi. The averages of the BMS scores were consistently higher in the Tranexamic acid-treated mice when compared to the vehicle-treated mice over time and became significantly higher at 7 dpi (t = 2.124 *p* = 0.038; Fig. 5d). At 28 dpi, six out of seven Tranexamic acid-treated mice achieved a BMS score of 5 (i.e. showed frequent or consistent plantar stepping). Moreover, two out of seven Tranexamic acid-treated mice acquired a mild trunk stability. The BMS scores of vehicle-treated mice were between 3 to 5. All vehicle-treated mice showed a severe trunk instability such as leaning, waddling or near collapse of the hindlimbs and three out of eight did not perform a frequent plantar stepping. The average BMS subscores were consistently higher in the Tranexamic acid-treated mice than in vehicle-treated mice from 7 to 28 dpi, becoming significantly higher from 21 (*t* = 3.359, *p* = 0.001; Fig. 5e) to 28 dpi (*t* = 4.776, *p* < 0.0001; Fig. 5e).

### Tranexamic acid reduces the extent of the lesion after a T9 contusion injury

**Figure 6:**
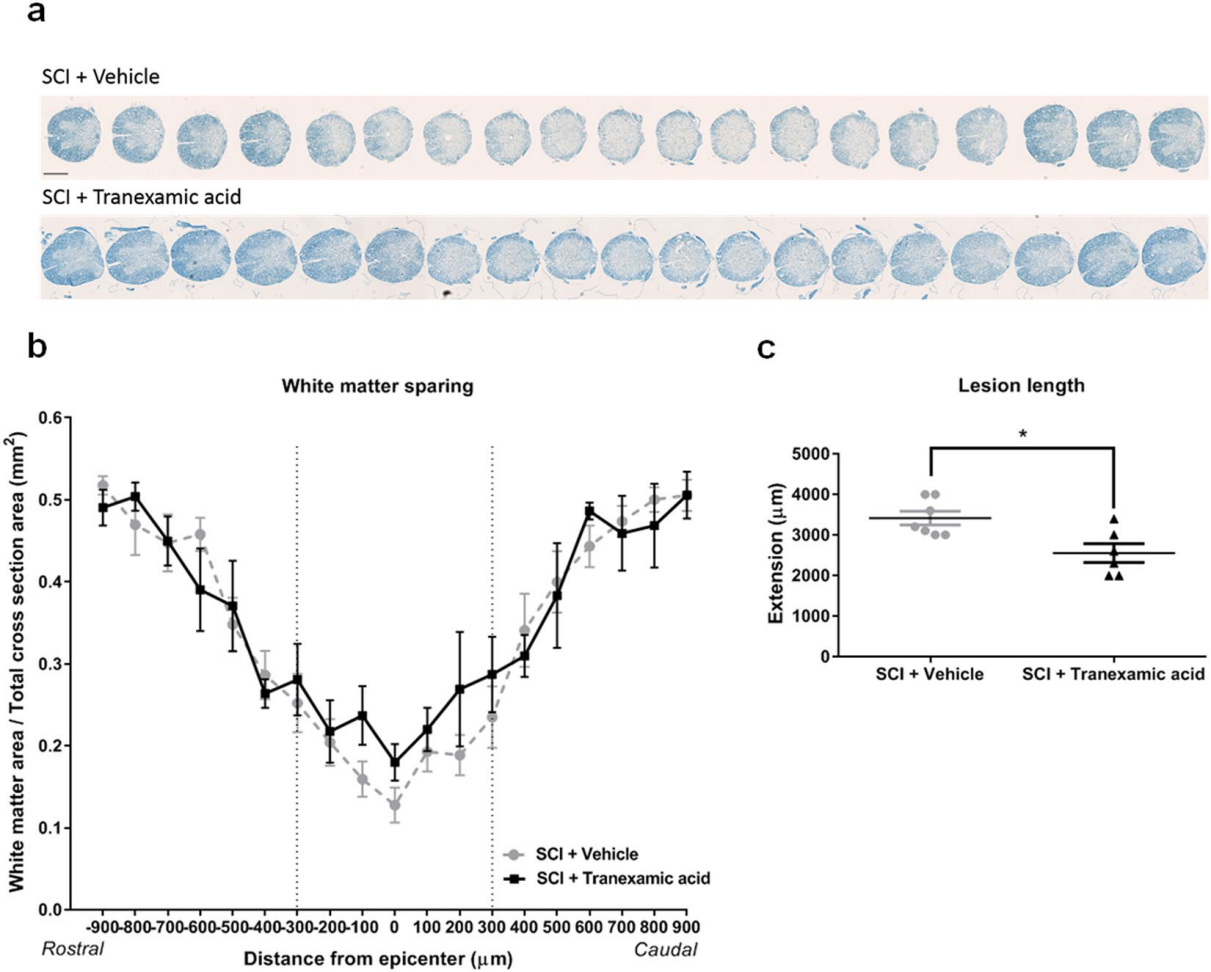
White matter sparing and lesion length in vehicle-treated mice and Tranexamic acid-treated mice at 28 days after SCI. (**a**) Representative spinal cord sections of vehicle-treated mice comparing to Tranexamic acid- treated mice labelled with luxol fast blue staining. Scale bar: 500 µm. (**b**) The white matter area per total cross section area from the epicenter to 900 µm on the rostral and caudal sides in vehicle-treated mice and in Tranexamic acid-treated mice. Two-way ANOVA followed by Bonferroni post hoc correction. n = 7 for SCI+ Vehicle and n = 6 for SCI+ Tranexamic acid. (**c**) Lesion length in vehicle-treated mice (n = 7) and in Tranexamic acid-treated mice (n = 6). *p < 0.05, Student’s t-test with Welch’s correction.

To infer about the demyelination status upon injury, we compared the spared white matter area per total cross section area between the Tranexamic acid-treated mice and vehicle-treated mice using a modified luxol fast blue staining (Fig. 6a,b). The averages of spared white matter per total cross section area in the Tranexamic acid-treated mice were not significantly but consistently larger than in the vehicle-treated mice over a 300 µm rostrally and caudally from the epicentre of the lesion (Fig. 6b). Furthermore, we also evaluated the length of the lesion by counting the number of consecutive sections with a visible lesion. This analysis demonstrated that vehicle-treated mice had lesion lengths significantly higher than Tranexamic acid-treated mice (*t* = 3.019, *p* = 0.0136; Fig. 6c). The white matter area was better preserved, and the extent of the lesion was smaller in Tranexamic acid-treated mice.

## Discussion

The zebrafish larva is a particularly versatile vertebrate model for investigating novel functions of small-molecule compounds and provides a platform for straightforward and cost-effective drug screenings. Moreover, drug repurposing, the testing of approved drugs for new off-label effects, promises to accelerate the rapid translation of hits from zebrafish chemical screens to clinical trials^17^.

In line with these notions, we developed a phenotypic assay for spinal cord injury (SCI) indication. With this assay, we were able to screen small molecules from an FDA-approved chemical compounds library using 5 dpf zebrafish larvae with complete transected spinal cords. As zebrafish larvae regenerate the spinal cord, our approach intended to find molecules that would accelerate the regenerative process, i.e. in which we could detect a locomotor improvement at a time-point where recovery was not expected.

Several studies describe the dynamics of spinal cord recovery after injury using zebrafish larvae^21,25,33,34^. What emerges from these studies is that the time-course of events depend on the type of injury, the larval stage at which the injury is performed and ultimately on the operator. Thus, we first needed to describe the time-course of the recovery dynamics in our larval zebrafish transected spinal cord model.

In our model, we demonstrate that the wound closes within 48 hours and the resected tissues seem to be completely recovered within 6 days.

From 1 to 4 dpi, injured larvae showed locomotor impairments when compared to uninjured larvae, as measured by total distance moved and turn angle. At 6 dpi, the injured larvae had significantly recovered their locomotor function, reaching levels equivalent to the uninjured ones.

Moreover, at the lesion site, injured larvae: (1) initiated the formation of the glial bridge between 2–3 dpi; (2) started to show increased numbers of HB9^+^ motor neurons at 2 dpi; and (3) revealed an evident regeneration of HB9^+^ peripheral axons between 3–4 dpi. Overall, our data demonstrate that a robust motor neuron and functional regeneration only occurs at 6 dpi.

Taking the time-course of the spinal cord recovery dynamics in our model, we decided to perform a phenotypic screen where chemical compounds were administered at 1 dpi (a time-point where none of the cellular features mentioned above is detected) and locomotor assessment was carried out at 2 dpi (a time-point where there is no functional recovery).

We are confident of the power of our zebrafish larval SCI model and the way we designed our screen. We were able to show the locomotor rescue properties of Dopamine, Riluzole and Minocycline, three different pharmacological classes of molecules with proved functional recovery in SCI animal models^1,12–14,30,31^. Notably, one of the blinded selected candidates from the library that rescued motor impairments in our SCI model was D-Cycloserine, a molecule that turned out to be in the pipeline for a phase IV clinical trial for SCI indication. These findings are particularly important as a strong proof-of-concept for the screen described here.

In our phenotypic screening assay, 44 of the screened molecules significantly improved motor function. As the small molecules used in this screen are FDA approved, we took advantage of previous studies and reports, first to exclude the compounds already described for the indication of SCI and then to evaluate associated toxicity, pharmacokinetic/pharmacodynamic properties and possible mechanisms of action. We found Tranexamic acid as one of the most promising candidates from the 3 of final selected hits with rescuing motor impairment properties (in both total distance moved and turn angle parameters) and spinal cord recovery potential. Interestingly in zebrafish, the turn angle parameter was recently associated with demyelination and inability to control motor direction robustly^35^. In accordance to this, our data suggests that Tranexamic acid improves not only the swimming ability but also motor direction control.

One limitation of our screen is the use of a single concentration of 25 µM during a short period of 24 hours, that can lead to an erratic exclusion of compounds with no activity at this concentration. We had to do a compromise between efficacy, time and cost control. Therefore, we chose one single application at the highest concentration commonly used for drug screens in zebrafish^17^. Another limitation is the exclusion of molecules that do not cross the BBB, that could have a positive effect at least in earlier phases of the SCI condition, when the BBB is disrupted.

We next tested the efficacy of Tranexamic acid in a non-regenerative vertebrate model, to evaluate its potential as a therapeutic for SCI in mammals, thus further validating our zebrafish screen platform.

Daily administration of Tranexamic acid during the first 8 days after injury, significantly promoted an improvement of locomotor behaviour and reduced the extent of the lesion. Tranexamic acid is known to act as a pro-coagulant. We propose that, in the context of SCI, it could limit the toxic effect of the parenchymal haemorrhage and in this way control the extension of the lesion, that would then lead to a better locomotor outcome. Recently, it was demonstrated that a combinatorial therapy (Tranexamic acid plus a second pro-coagulant plus an osmotic agent) reduces the extent of the lesion in the acute phase of SCI in rats^4^, further strengthening our results. Importantly, this later study does not report the state of the lesion at time-points later than 24 hours, neither correlates the lesion extension with neurobehavioral outcomes. We believe that our findings will be relevant for humans, as it was shown that the extent of parenchymal haemorrhage is also correlated with a poor functional outcome in SCI patients^4,36^.

Overall, this study highlights the predictive value of the zebrafish larva as a drug screening model for SCI. We present a simple, fast and automated phenotypic screening with proved efficacy in a rodent T9 contusion model of SCI.

The administration of Tranexamic acid during the acute phase seems to have a protective effect, possibly reducing the secondary damage. Further investigations are needed before translation to humans, namely, to evaluate the minimal effective dose, the optimal administration scheme and to further unveil the mechanism of action, clarifying the effect of this potential therapeutic on neuroprotection and neuroregeneration after SCI.

Ultimately, our new approach, as opposed to the quest for new drugs that require long processes of validation, can lead to a faster translation to humans.

## Methods

### Ethics statement

All experiments involving animals were performed in accordance with the European Community guidelines (Directive 2010/63/EU), the Portuguese law on animal care (DL 113/2013) and were approved by Instituto de Medicina Molecular Internal Committee (ORBEA) and the Portuguese Animal Ethics Committee (DGAV). All efforts were made to minimize the number of animals used and to decrease suffering of the animals used in the study.

### Animals

Tg(*mnx1*:GFP^m12^), abbreviated as *hb9*:GFP were maintained and bred in constant conditions, by following standard guidelines for fish care and maintenance protocols^37^. Male and female fish were used in the experiments. Adult female C57BL/6J mice (12-weeks-old; Charles River) were used in this study. The mice were housed three to four per cage and were maintained on a 12-hour light/dark cycle with food and water *ad libitum* for the duration of the study.

### Zebrafish larval spinal cord transection injury

Zebrafish larvae with 5 days-post-fertilization (dpf) were anesthetized in 0,02% MS222 (Sigma-Aldrich) and placed in a lateral position on a petri dish under a stereoscope. Transection injuries were performed at the level of the anal pore, using a sharp 28-gauge injection needle, while leaving the notochord and majors vessels intact. After the injuries, larvae were transferred to a new petri dish with embryo medium (EM [5 mM NaCl, 0.17 mM KCl, 0.33 mM CaCl_2_, 0.33 mM MgSO_4_, pH 7.4]).

### Mouse spinal cord injury and post-operative care

An Infinite Horizon Impactor (PSI) was used to perform a contusion-type of injury on adult C57BL/6 female mice^38^. Briefly, under deep anesthesia with Ketamine and Xylazine (90 mg/kg and 10 mg/kg respectively, i.p.) mice received a dorsal laminectomy at the T9 level. All surgeries were performed under aseptic conditions. A controlled force-defined impact at 75 kdynes (moderate to severe contusion) was delivered to the exposed cord with a stainless-steel impactor tip after securing the lateral processes of T8 and T10. In this study, an animal was excluded when the actual force after impact was >2 SD 75 Kdynes or if the actual displacement value of the impactor was outside the interval 500–700 µm ± SD. The mice were then injected subcutaneously with 0,5 ml of sterile saline daily for a week. The mice underwent gentle bladder expression twice daily until the end of the study or until they were voiding on their own. Weight was monitored daily until 8 days-post-injury (dpi) and then weekly for the duration of the study (28 dpi). A 10% weight loss was typically observed after injury, and a high caloric pellet (Supreme Mini-Treats™ S05478 and S05472) was provided as a dietary supplement.

### Small molecule library

The PHARMAKON 1600 library is a collection of 1600 known drugs from US and International Pharmacopeia. The compounds have reached clinical evaluation and demonstrated biological activity against known targets. Many of the drugs represented in the library are available on the market. The library was purchased from MicroSource Discovery Systems, Inc. (USA) and compounds were delivered in microplates (10 mM, dissolved in DMSO) and kept at −80 °C until use.

### Small molecule administration

#### In a spinal cord injury zebrafish larval model

An optimized protocol for zebrafish exposure to chemical compounds was developed and then used to test therapeutic controls and also to screen a pool of compounds from a library of small molecules (PHARMAKON 1600). Zebrafish larvae were allowed to develop in EM with 1 µM methylene blue until 5 days-post-fertilization (dpf). At 1 dpi, larvae were randomly distributed into 6-well plates containing EM+ 10 mM HEPES and the chemical compounds were added to the medium at the desired concentration. For each experiment, uninjured larvae and injured larvae treated with 1% DMSO (a concentration that does not affect animal behaviour (were used as vehicle controls and injured larvae treated with Minocycline (M9511, SIGMA-Aldrich) were used as therapeutic positive controls. The small molecules from the library were tested at 25 µM per condition. Minocycline was tested at the same concentration as the library molecules. Riluzole (from the small molecules library) was tested at 0,5 µM and Dopamine (H8502-5G, SIGMA-Aldrich) was tested at 30 µM.

#### In a spinal cord injury mouse contusion model

The treatment dosages (equivalent to the human market dose^39^ were aliquoted using a coded system to maintain double-blinded measures. The code was unveiled only after all the behavioural tests were completed. Mice were randomly distributed to each experimental groups (SCI+ vehicle and SCI+ small molecule treatment). The small molecule and vehicle were administered via i.p., beginning 1 hour after injury, and then daily until 8 dpi.

### Zebrafish larvae behavioural analysis

After 1 dpi, injured *hb9*:GFP larvae were exposed during 24 hours to the chemical compounds added to the medium. Uninjured larvae treated with vehicle (1% DMSO) (n = 16 larvae), injured larvae treated with vehicle (1% DMSO) (n = 13–16 larvae) and injured larvae treated with Minocycline as the positive control (n = 16 larvae) were tested concomitantly. Behavioural assessment was performed at 7 dpf (i.e. 2 dpi with 24 hours of chemical treatment) using DanioVision™ (Noldus Information Technology, the Netherlands) automated tracking system for zebrafish larvae. Larvae were allowed to freely swim in a 96-well plate (1 larva/well) with EM+ 10 mM HEPES and their swimming activity was tracked for 90 minutes, under 10 minutes light-dark cycles (i.e. 3 light cycles and 3 dark cycles). The acquired track data was analysed using the Ethovision X.T. 8.5.614 software (Noldus, Wageningen, Netherlands). Only swimming activity obtained in the 3 dark periods (i.e. under infrared light) were analysed^40^. The parameters analysed were total distance moved (mm) and turn angle (represents the change of head direction, a measure of the fitness of the movement) (Fig. 3a,b).

### Basso mouse scale (BMS) test for open-field locomotion

Open-field locomotion was assessed with the BMS rating system, which allows the reliable measurement of hindlimb recovery in mice following SCI^41^. The open-field used was a round platform with 85 cm in diameter and 30,5 cm high, located in a quiet testing room with normal lighting. The mice were acclimated to the testing table for 5 minutes daily for 2 weeks before surgery. Before surgery, the mice were tested to obtain baseline pre-operation locomotion values, showing the expected maximum BMS score of 9. After injury, the BMS score of each mouse was evaluated at 1, 3, 7, 14, 21 and 28 days to determine the functional recovery after treatment. The BMS score evaluation was always performed by two investigators who were blind to the treatment groups and scored hindlimb locomotion for 4 minutes per mouse. If the scores differed between raters, the final score will be the average of both scores. To summarize BMS testing, the timer was initiated when the mouse was placed in the open-field and we first decided whether the mouse did plantar stepping. If there was plantar stepping, then the frequency of stepping and coordination was evaluated. If not, then ankle movement of dorsal stepping was evaluated and appropriately scored.

For the mice that achieved a threshold of frequent stepping (i.e. BMS score of 5), we initiated BMS subscoring by quantifying the improvement on stepping frequency, coordination, paw position, trunk stability, and tail position.

### Whole mount immunostaining and confocal microscopy

Zebrafish larvae were fixed overnight at 4 °C in 4% paraformaldehyde (PFA). Larvae were then gradually dehydrated to methanol 100% and stored at −20 °C. For whole mount immunostaining, larvae were gradually rehydrated to phosphate buffer saline (PBS). The tissue was then permeated in 100% acetone for 15 minutes at −20 °C, washed with 0.5% PBS-Triton X-100 and blocked in blocking solution (1% bovine serum albumin in PBS with 1% DMSO and 0.05% Triton X-100) for 2 hours at room temperature. Whole mount tissues were incubated overnight at 4 °C with anti-GFP (1:1000, A6455); anti-GFAP (1:200, zrf-1, Zirc) primary antibodies, washed in 0.1% PBS-Triton X-100, PBS and re-incubated overnight at 4 °C with AlexaFluor 568 (1:1000, A11004) or AlexaFluor 488 (1:1000, A11008) secondary antibodies. After staining, larvae were washed in PBS and flat-mounted on a fluorescent mounting medium with DABCO, under a stereoscope. Z-stack compositions were acquired in a confocal microscope (Zeiss LSM 880 META, Carl Zeiss MicroImaging, Göttingen, Germany) with 20× magnification. Cell content was assessed by counting the number of Hb9^+^ cells from maximum intensity projections with Image J software and by measuring the length of motor neurons axons that cross the lesion site.

### Tissue processing

At the end of behaviour tests, mice were transcardially perfused with saline, followed by 4% paraformaldehyde in 0,1 M phosphate-buffered saline (PBS) at pH 7,4. After an overnight rinse in phosphate buffer and cryoprotection for 3 days in 30% sucrose, spinal cords were frozen in optimal cutting temperature (OCT) compound (Sakura Finetek USA) in blocks from 3 mm rostral to the injury epicentre to 3 mm caudal (6 mm total). Each block was cut on a cryostat in 10 μm transverse sections, mounted on slides in 10 alternating sets, and stored at −20 °C until they were stained^42^.

### Modified luxol fast blue staining and white matter sparing analysis

One set of sections spaced 100 μm apart and spanning the entire block was stained with modified luxol fast blue staining. The epicentre was identified as the section of tissue with the smallest area of blue-stained white matter in the rim.

NanoZoomer SQ (Hamamatsu) was used to measure the cross-sectional area of white matter sparing (WMA) and the total cross-sectional area of the tissue section (TCA), and then the proportional cross-sectional area at the lesion epicentre was calculated (WMA/TCA). The rostral and caudal extents of each lesion were determined by inspection, and lesion length was calculated by multiplying the number of sections containing lesioned tissue by the distance between each section (100 μm). All lesion analysis was done with coded sections and by an investigator unaware of treatment or outcome groups.

### Statistical analysis

Data analysis from larval zebrafish SCI model and all graphical representations were performed using Prism 8 software (GraphPad Software, Inc., San Diego, CA, USA). Statistical tests used were two-tailed. Mean comparisons between the different groups were performed using unpaired Student’s t-test with Welch’s correction. All data are expressed as the mean ± standard error of the mean (SEM). Data from the BMS were analysed using repeated measures two-way analysis of variance (ANOVA) followed by Bonferroni’s *post-hoc* test using SigmaPlot 14. P value < 0.05 was considered significant.

## Supplementary information

Supplementary Information
