# Author Correction: A zebrafish drug screening platform boosts the discovery of novel therapeutics for spinal cord injury in mammals

**DOI:** 10.1038/s41598-020-57443-7

**Published:** 2020-01-15

**Authors:** Diana Chapela, Sara Sousa, Isaura Martins, Ana Margarida Cristóvão, Patrícia Pinto, Sofia Corte-Real, Leonor Saúde

**Affiliations:** 1 TechnoPhage, SA, Av. Prof. Egas Moniz, 1649-028 Lisboa, Portugal; 2 Instituto de Medicina Molecular e Instituto de Histologia e Biologia do Desenvolvimento, Faculdade de Medicina da Universidade de Lisboa, 1649-028 Lisboa, Portugal; 3 Instituto de Medicina Molecular, Faculdade de Medicina da Universidade de Lisboa, 1649-028 Lisboa, Portugal

Correction to: *Scientific Reports* 10.1038/s41598-019-47006-w, published online 19 July 2019

This Article contains an error in the Introduction.

“Recently, the intravenous administration of Minocycline showed to reduce cell death and improve hindlimb function in mammal SCI models^13,14^.”

should read:

“The administration of Minocycline showed to reduce cell death and improve hindlimb function in mammal SCI models^13,14^.”

Additionally, some of the methods presented in this Article were previously developed by different Authors. The Authors of this Article neglected to cite the previous papers, which are included below as Reference 1 and 2 and should be cited in the methods section as below:

**Mouse spinal cord injury and post-operative care**

[…] A controlled force-defined impact at 75 kdynes (moderate to severe contusion) was delivered to the exposed cord with a stainless-steel impactor tip after securing the lateral processes of T8 and T10^1^. […] A 10% weight loss was typically observed after injury, and a high caloric pellet (Supreme Mini-Treats™ S05478 and S05472) was provided as a dietary supplement^1^.

**Basso mouse scale (BMS) test for open-field locomotion**

Open-field locomotion was assessed with the BMS rating system, which allows the reliable measurement of hindlimb recovery in mice following SCI^1,^^41^. […] If there was plantar stepping, then the frequency of stepping and coordination was evaluated. If not, then ankle movement of dorsal stepping was evaluated and appropriately scored^1^. […]

**Modified luxol fast blue staining and white matter sparing analysis**

[…] All lesion analysis was done with coded sections and by an investigator unaware of treatment or outcome group^2^.
