# Supplementary material for: A zebrafish drug screening platform boosts the discovery of novel therapeutics for spinal cord injury in mammals
Source: Sci Rep. 2019 Jul 19;9:10475. doi: 10.1038/s41598-019-47006-w (PMC6642202; doi:10.1038/s41598-019-47006-w)
Supplement: Supplementary file 1 — Supplementary Information [file 41598_2019_47006_MOESM1_ESM.pdf]

A zebrafish drug screening platform boosts the discovery of novel therapeutics for spinal cord injury in mammals

Diana Chapela[1,3], Sara Sousa[1+], Isaura Martins[3+], Ana Margarida Cristóvão[3], Patrícia Pinto[1], Sofia Corte-Real[1] and Leonor Saúde[2*]

1. TechnoPhage, SA, Av. Prof. Egas Moniz, 1649-028 Lisboa, Portugal
2. Instituto de Medicina Molecular e Instituto de Histologia e Biologia do Desenvolvimento, Faculdade de Medicina da Universidade de Lisboa, 1649-028 Lisboa, Portugal
3. Instituto de Medicina Molecular, Faculdade de Medicina da Universidade de Lisboa, 1649-028 Lisboa, Portugal
*msaude@medicina.ulisboa.pt
[+]these authors contributed equally to this work

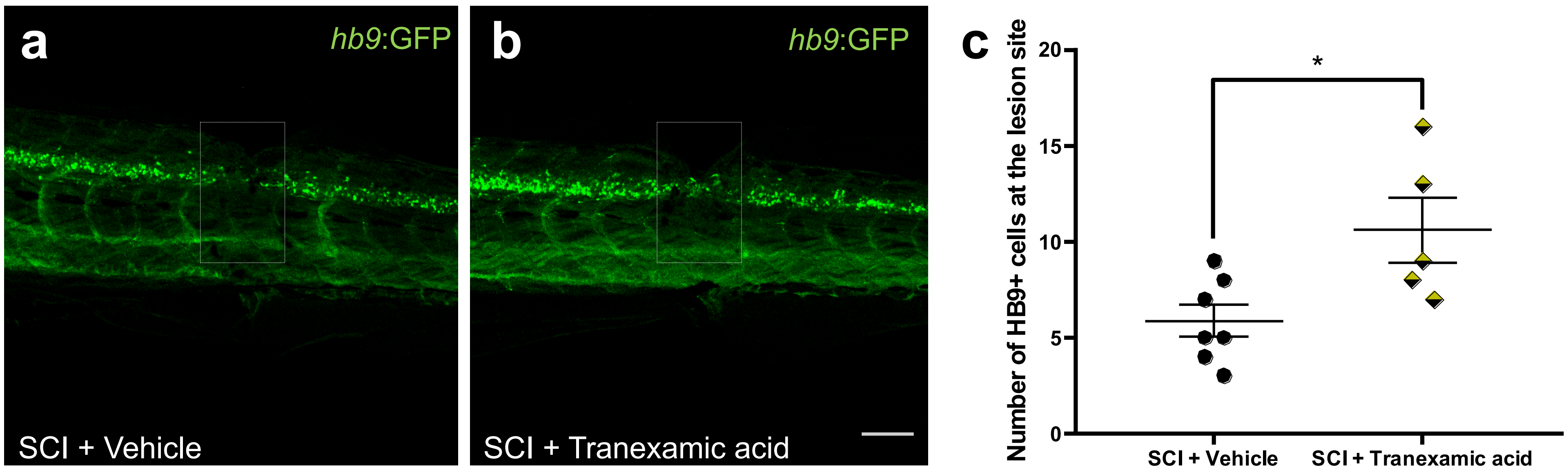

Supplementary Figure S1. Labeling pattern of injured *Hb9*:GFP transgenic larvae treated with vehicle **(a)** and Tranexamic acid **(b)** with immunohistochemistry against GFP (green) to reveal HB9⁺ motor neurons at the lesion site. Rostral side is to the left and dorsal side is up. The rectangle marks the injury site. Scale bar: 100 µm. **(c)** Number of HB9⁺ motor neurons at the lesion site of injured larvae treated with vehicle (n=7) comparing to injured Tranexamic acid-treated larvae (n=5). *p<0.05, Student´s t-test with Welch's correction.

Supplementary Table S1

Descriptive values and statistics of the larvae locomotor behaviour data underlying the analysis shown in Figure 1.

Total distance moved			Turn angle		
	**Uninjured**	**1 dpi**		**Uninjured**	**1 dpi**
**Number of values**	16	16	**Number of values**	16	16
**Minimum**	24,05	3,95	**Minimum**	47,36	108,2
**Maximum**	166,3	72,19	**Maximum**	164,6	310,6
**Range**	142,2	68,24	**Range**	117,2	202,3
**Mean**	100	27,28	**Mean**	100	212,4
**Std. Deviation**	39,57	18,94	**Std. Deviation**	40,33	58,93
**Std. Error of Mean**	9,893	4,735	**Std. Error of Mean**	10,08	14,73
	**Uninjured**	**2 dpi**		**Uninjured**	**2 dpi**
**Number of values**	16	16	**Number of values**	16	16
**Minimum**	66,82	2,218	**Minimum**	63,82	115,6
**Maximum**	156,3	112,4	**Maximum**	144,8	310,1
**Range**	89,45	110,1	**Range**	80,99	194,4
**Mean**	100	49,58	**Mean**	100	166,5
**Std. Deviation**	29,91	29,75	**Std. Deviation**	27,01	58,59
**Std. Error of Mean**	7,477	7,437	**Std. Error of Mean**	6,752	14,65
	**Uninjured**	**3 dpi**		**Uninjured**	**3 dpi**
**Number of values**	12	11	**Number of values**	12	11
**Minimum**	34,33	9,809	**Minimum**	43,57	72,57
**Maximum**	159,8	116,1	**Maximum**	182,3	247,6
**Range**	125,5	106,3	**Range**	138,8	175
**Mean**	100	59,03	**Mean**	100	159,4
**Std. Deviation**	40,02	36,61	**Std. Deviation**	42,51	51,76
**Std. Error of Mean**	11,55	11,04	**Std. Error of Mean**	12,27	15,61
	**Uninjured**	**4 dpi**		**Uninjured**	**4 dpi**
**Number of values**	16	16	**Number of values**	16	16
**Minimum**	40,03	18,51	**Minimum**	45,34	80,87
**Maximum**	159,1	133,7	**Maximum**	186,7	203,7
**Range**	119,1	115,2	**Range**	141,4	122,9
**Mean**	100	70,51	**Mean**	100	127,9
**Std. Deviation**	29,76	32,48	**Std. Deviation**	36,98	43,71
**Std. Error of Mean**	7,441	8,121	**Std. Error of Mean**	9,245	10,93
	**Uninjured**	**6 dpi**		**Uninjured**	**6 dpi**
**Number of values**	15	11	**Number of values**	15	11
**Minimum**	4,961	17,79	**Minimum**	56,19	66,98
**Maximum**	194,5	173,6	**Maximum**	252,2	232,3
**Range**	189,5	155,8	**Range**	196	165,3
**Mean**	100	101,4	**Mean**	100	116,4
**Std. Deviation**	53,14	46,54	**Std. Deviation**	48,11	51,11
**Std. Error of Mean**	13,72	14,03	**Std. Error of Mean**	12,42	15,41

Supplementary Table S2

Descriptive values and statistics of the larvae locomotor behaviour data underlying the analysis shown in Figure 4.

Total distance moved		SCI			Turn angle		SCI		
	Uninjured	Vehicle	Dopamine	Riluzole		Uninjured	Vehicle	Dopamine	Riluzole
Number of values	16	13	14	16	Number of values	16	13	14	16
Minimum	50,83	17,64	55,58	22,89	Minimum	52,56	87,44	93,7	73,88
Maximum	179,2	117,6	152,3	169,4	Maximum	164,8	193,1	158,9	171,2
Range	128,4	99,94	96,74	146,5	Range	112,3	105,6	65,19	97,36
Mean	100	71,59	95,19	106,6	Mean	100	131,6	136	114,5
Std. Deviation	35,59	30,81	27,53	41,14	Std. Deviation	37,14	32	20,04	25,5
Std. Error of Mean	8,898	8,545	7,359	10,29	Std. Error of Mean	9,285	8,875	5,355	6,376
		SCI					SCI		
	Uninjured	Vehicle	Minocycline			Uninjured	Vehicle	Minocycline	
Number of values	16	15	16		Number of values	16	15	16	
Minimum	39,87	10,57	37,9		Minimum	73,24	100,8	96,61	
Maximum	148,3	127,6	200		Maximum	152,3	259,3	167,1	
Range	108,4	117	162,1		Range	79,01	158,5	70,48	
Mean	100	54,8	107,5		Mean	100	151,6	126,3	
Std. Deviation	28,17	27,59	57,77		Std. Deviation	18,21	40,16	18,22	
Std. Error of Mean	7,042	7,124	14,44		Std. Error of Mean	4,553	10,37	4,556	
		SCI					SCI		
	Uninjured	Vehicle	D-Cycloserine			Uninjured	Vehicle	D-Cycloserine	
Number of values	16	16	16		Number of values	16	16	16	
Minimum	44,64	17,13	38,97		Minimum	75,94	121,3	87,65	
Maximum	219	112,5	274,2		Maximum	133,9	339,9	239	
Range	174,4	95,34	235,3		Range	57,93	218,6	151,4	
Mean	100	58,96	98,21		Mean	100	182,9	173,7	
Std. Deviation	45,41	31,54	65,41		Std. Deviation	17,91	66,04	39,39	
Std. Error of Mean	11,35	7,886	16,35		Std. Error of Mean	4,478	16,51	9,847	
		SCI					SCI		
	Uninjured	Vehicle	Tranexamic acid			Uninjured	Vehicle	Tranexamic acid	
Number of values	16	16	16		Number of values	16	16	16	
Minimum	47,51	21,46	9,794		Minimum	68,02	96,79	54,52	
Maximum	178,7	99,75	145,3		Maximum	155,9	180,4	202,4	
Range	131,2	78,29	135,5		Range	87,91	83,64	147,8	
Mean	100	58,76	84,27		Mean	100	136	102,1	
Std. Deviation	34,11	20,62	33,83		Std. Deviation	25,12	23,39	36,71	
Std. Error of Mean	8,527	5,154	8,457		Std. Error of Mean	6,281	5,846	9,179	
